# Synthesis and characterization of zinc oxide nanoparticles by using polyol chemistry for their antimicrobial and antibiofilm activity

**DOI:** 10.1016/j.bbrep.2018.11.007

**Published:** 2018-12-12

**Authors:** Pranjali P. Mahamuni, Pooja M. Patil, Maruti J. Dhanavade, Manohar V. Badiger, Prem G. Shadija, Abhishek C. Lokhande, Raghvendra A. Bohara

**Affiliations:** a Centre for Interdisciplinary Research, D.Y. Patil University, Kolhapur, India; b Department of Microbiology, Shivaji University, Kolhapur, India; c CSIR, National Chemical Laboratory, Pune, India; d Department of Materials Science and Engineering, Chonnam National University, Gwangju, Republic of Korea; e CURAM, Center for Research in Medical Devices, National University of Ireland Galway, Ireland

**Keywords:** Antibacterial, Antibiofilm activity, ZnO polyol method

## Abstract

The present investigation deals with facile polyol mediated synthesis and characterization of ZnO nanoparticles and their antimicrobial activities against pathogenic microorganisms. The synthesis process was carried out by refluxing zinc acetate precursor in diethylene glycol(DEG) and triethylene glycol(TEG) in the presence and in the absence of sodium acetate for 2 h and 3 h. All synthesized ZnO nanoparticles were characterized by X-ray diffraction (XRD), UV visible spectroscopy (UV), thermogravimetric analysis (TGA), fourier transform infrared spectroscopy (FTIR), field emission scanning electron microscopy(FESEM), transmission electron microscopy (TEM) and energy dispersive X-ray spectroscopy (EDX) technique. All nanoparticles showed different degree of antibacterial and antibiofilm activity against Gram-positive *Staphylococcus aureus* (NCIM 2654)and Gram-negative *Proteus vulgaris* (NCIM 2613). The antibacterial and antibiofilm activity was inversely proportional to the size of the synthesized ZnO nanoparticles. Among all prepared particles, ZnO nanoparticles with least size (~ 15 nm) prepared by refluxing zinc acetate dihydrate in diethylene glycol for 3 h exhibited remarkable antibacterial and antibiofilm activity which may serve as potential alternatives in biomedical application.

## Highlights

• Synthesis of Zno NPs of different size & shape by tuning polyol/catalyst/reaction time.
• Shape and size control were possible by varying these parameters.
• Antibacterial and antibiofilm activity were studied against Staphylococcus aureus and Proteus vulgaris.
• Comparative study revealed DEG synthesis for 3 h in absence of sodium acetate showed maximum antibacterial/biofilm activity.

## 1 Introduction

Biofilms are the complex communities of microorganisms attached to any biological or non-biological surface that remain enclosed in self-produced hydrated polymeric matrix [1], [2]. Microorganisms in biofilm transcribe genes that are different from the genes transcribed by planktonic bacteria [3]. The cells in the biofilm are inherently protected from phagocytosis, develops high resistance to antibiotics which make them difficult to treat [4], [5], [6], [7]. Both Gram-positive and Gram-negative bacteria can form the biofilm on various medical devices such as catheters, prosthetic joints, endotracheal tubes, heart valves, contact lenses and ortho-dental instruments [8]. In this regard, *Staphylococcus aureus* and *Proteus vulgaris* are biofilm-forming pathogens on medical implants able to produce severe biofilm-associated infections such as urinary tract infection, musculoskeletal infection and respiratory tract infection [9]. It has been estimated that the maximum bacterial infections treated in hospitals are associated with bacterial biofilm [6]. In fact, the number of implant-associated infections near about 1 million/year in the US alone and their direct medical costs exceed $3 billion annually [10].

The problem of biofilm-related infections could be resolved by removal of biofilm physically or removal of implants which is not feasible economically. Other methods like use of depolymerase enzyme and the use of bacteriophages could be used to control biofilm formation [11]. Recent reports suggest that several synthesized antimicrobial peptides (AMPs) are able to interact with the membrane through penetration or dissolving the biofilms [12], [13]. Alternatives to these conventional methods which recommend, recent developments in nanotechnology that have been proven to be an efficient approach to control biofilm formation [14].

The ability of nanomaterials for biofilm disruption has been reported. For example, Simona and Prodan et al investigated the effect of glycerol iron oxide nanoparticles for biofilm inhibition produced by *Pseudomonas aeroginosa* [15]. Among nanosized metal oxides, zinc oxide (ZnO) has gained much more attention due to its interesting properties such as high surface to volume ratio, low cost and long-term environmental stability [16], [17]. According to Sirelkhatim et al. and Dhillo et al., it is already reported by several studies that ZnO nanoparticles are non-toxic to human cells and toxic to bacterial cells. Toxicity studies showed that DNA in human cells do not get damaged by zinc ions. This fact made ZnO nanoparticles biocompatible to human cells [16], [18], [19].

Various methods have been used to prepare zinc oxide nanoparticles suchas hydrothermal [20], [21], [22], [23], solvothermal methods [24], [25],microemulsion [26], sol-gel [27], [28] and thermal decomposition of precursors [29], [30].

According to Raghupathi et al. and Applerot et al., ZnO nanoparticles exhibit a maximum degree of antibacterial activity with the decrease in particle size [7], [31]. Method of synthesis of nanoparticles strongly affects the size and shape of nanoparticles, which determines the properties of nanoparticles [32], [33].

Fievet, Lagier, and Figlarz first introduced the use of polyols for the synthesis of small particles termed as “polyol process” or “polyol synthesis.” The polyol synthesis allows the formation of ZnO nanoparticles with excellent crystalline quality and controlled morphology. Its peculiarity lies in the properties of polyols like high boiling point (up to 320 °C), high dielectric constant, the solubility of simple metal salt precursors and coordinating properties for surface functionalisation preventing agglomeration [34], [35]. Also, the presence of weak base sodium acetate in the reaction controls the nucleation process and assembly process through which nanoparticles with different morphology can be obtained [36].

In the present investigation, we have synthesized ZnO nanoparticles by applying different approaches, (i) regular synthesis in polyols, (ii) in presence of sodium acetate, (iii) increasing reaction time. We have employed different strategies to synthesize ZnO nanoparticles. The synthesis method mainly involves reflux of zinc acetate dihydrate precursor in diethylene glycol (DEG) and triethylene glycol (TEG) in the presence and in absence of weak base sodium acetate for varied reaction time. The effect of these two polyols, presence and absence of sodium acetate and reaction time on size and morphology of synthesized ZnO nanoparticles is presented. These nanoparticles were studied for their antimicrobial and antibiofilm activity against *Staphylococcus aureus* (NCIM 2654) and *Proteus vulgaris* (NCIM 2813).

## 2 Materials and methods

### 2.1 Materials

All chemicals used here were of analytical grade and used without further purification. All chemicals were purchased from Loba fine chemicals, Mumbai, India. The media have been procured from Himedia Laboratories Pvt. Ltd, Mumbai, India. Distilled water was used in the all experiments. The microorganisms, Gram-positive (*Staphylococcus aureus* NCIM 2654) and Gram-negative (*Proteus vulgaris* NCIM 2613) were collected from the National Collection of Industrial Microorganisms (NCIM), Pune, India.

### 2.2 Synthesis of ZnO nanoparticles

ZnO nanoparticles were prepared by refluxing precursor zinc acetate dihydrate (0.1 M) in diethylene glycol and triethylene glycol at 180 °C and 220 °C respectively. Reaction time varied for 2 and 3 h with and without sodium acetate (0.01 M). Before refluxing, the solution was kept on a magnetic stirrer at 80 °C for 1.5 h. After completion of reflux action, the samples were centrifuged at 8000 rpm for 15 min and washed with distilled water and ethanol for three times. Further, it was dried at 80 °C for overnight (Table 1, Table 2).

**Table 1:** Reaction conditions used for synthesis of Zinc oxide nanoparticles.

Polyol used	Sample ID	Zinc acetate dihydrate	Sodium acetate	Hydration ratio		Reaction time and temperature
DEG	A	0.1 M	–	2	All samples	2 h at 180 °C
DEG	B	0.1 M	0.01 M	2	Were	2 h at 180 °C
DEG	C	0.1 M	–	2	Kept on	3 h at 180 °C
DEG	D	0.1 M	0.01 M	2	Magnetic	3 h at 180 °C
TEG	E	0.1 M	–	2	Stirrer	2 h at 220 °C
TEG	F	0.1 M	0.01 M	2	at 80°C for	2 h at 220 °C
TEG	G	0.1 M	–	2	1 h	3 h at 220 °C
TEG	H	0.1 M	0.01 M	2		3 h at 220 °C

**Table 2:** Calculated crystallite size of ZnO NPs are listed below.

ZnO samples	Crystallite size from XRD in nm
DEG 2 h	~ 22 nm
DEG 2 h with sodium acetate	~ 23 nm
DEG 3 h	~ 15 nm
DEG 3 h with sodium acetate	~ 18 nm
TEG 2 h	~ 20 nm
TEG 2 h with sodium acetate	~ 21 nm
TEG 3 h	~ 18 nm
TEG 3 h with sodium acetate	~ 18 nm

Where, D = crystallite size, λ = X-ray wavelength, β = FWHM of diffraction peak and θ = .

angle of diffraction.

**Table 3:** TGA results of ZnO samples (1) DEG 2 h, (2) DEG 2 h with sodium acetate, (3) DEG 3 h, (4) DEG 3 h with sodium acetate, (5) TEG 2 h, (6) TEG 2 h with sodium acetate, (7) TEG 3 h, (8) TEG 3 h with sodium acetate.

	**(1)**	**(2)**	**(3)**	**(4)**	**(5)**	**(6)**	**(7)**	**(8)**
Initial weight	100	100	100	100	100	100	100	100
1st decomposition	168	190	147	162	170	197	192	184
2nd decomposition	480	486	457	480	495	485	484	460
%weight loss	4.7%	6.5%	4.5%	5%	4.8%	9.7%	9.4%	2.6%
Remaining residue	95.21	93.5	94.5	95	94.2	90.3	90.6	97.4

% weight loss and remaining residue for all ZnO samples are given in Table 3. From table listed above it was observed that, DEG 3 h(3) and TEG 3 h with sodium acetate (8) shows minimum weight loss and maximum final residue.

### 2.3 Reaction mechanism of ZnO formation

By considering the chemicals involved in the hydrolysis process, the mechanism of the ZnO nanoparticles formation is proposed as follows.

(1) *n*Zn (CH_3_COO_2_)·2H_2_O *n* + nDEG /TEG→[Zn(OH)_2_-DEG /TEG]n

(2) [Zn(OH)_2_-DEG /TEG]n→ (ZnO-DEG/TEG) + H_2_O

**Scheme 1:**
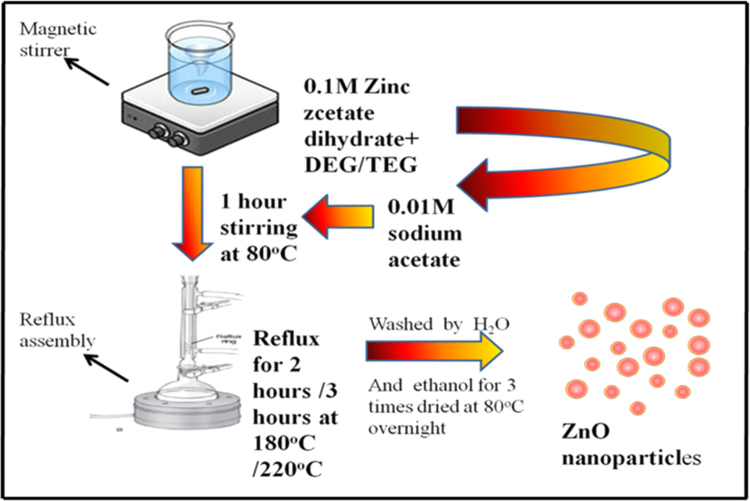
Schematic representation of synthesis of DEG and TEG mediated ZnO nanoparticles.

Formation of metal oxides proceeds in 2 steps: hydrolysis reaction and condensation reaction. Hydrolysis reaction is water dependent, absence of water in the reaction leads into failure of occurrence of next step of reaction that is condensation reaction which will not form any product. Also, due to presence of excess amount of water, particles start to agglomerate and give large sized particles with large distribution. So the hydrolysis ratio is considered as an important factor which affects the size and morphology. (Scheme 1).

Hydrolysis ratio is the ratio of number of moles of metal ions to number of moles of water. Alkaline ratio also considered an important factor affecting size and morphology. Amel Dalklaoui et al reported the effect of increasing alkaline ratio on morphology which showed the change in morphology from irregular and anisotropic forms to spherical form. Alkaline ratio is the number of moles of sodium hydroxide to metal which is attributed to the effect of OH^-^ ions on morphology. Also the concentration of precursor and temperature of the reaction affects the morphology of particles. In the present investigation, concentration of precursor, hydrolysis ratio and alkaline ratio is kept constant throughout the all synthesis processes of ZnO.

First, the reaction between zinc acetate dihydrate and DEG/TEG leads to esterification that forms (Zn-OH)_2_. Further dehydration of (Zn-OH)_2_ results into formation of ZnO nanoparticles. The basic approach for addition of sodium acetate was the addition of excess acetate ions that gives different particle morphologies than the particles synthesized in absence of sodium acetate. Sodium acetate causes a weak hydrolyzation, which controls the release rate of OH^−^ [36], [37], [38], [39], [40], [41], [42].

### 2.4 Characterization of nanoparticles

The X-ray diffraction studies of ZnO NPs were carried out using Rigaku 600Miniflex X-ray diffraction instrument (XRD) with Cukα radiation (λ = 1.5412 Å) in the scanning range of 10^0^-80^0^. To confirm the absorbance of ZnO NPs and to observe the changes in the absorbance caused due to variations in reaction conditions, UV–visible (UV–vis) spectra were carried in the wavelength range of 200–600 nm using Agilent Technologies Cary 60 UV–vis. In order to identify the characteristic functional groups present on the surface of the ZnO, Fourier transform infrared (FTIR) spectra of all samples were recorded by using JASCO INC 410,Japan,in a range of 400–4000 cm^−1^. Thermal gravimetric analysis(TGA) was carried out to observe thermal stability of ZnO on instrument PerkinElmer STA-5000. All samples were heated from 50 to 900 °C at the rate of 10 °C/min. The surface morphology of all synthesized ZnO were studied by field emission scanning electron microscopy(FESEM) and transmission electron microscopy(TEM). Elemental analysis was performed by energy dispersive X-ray (EDX) spectroscopy (JSM-6701F, JOEL, Japan).

### 2.5 The antimicrobial assay

Antimicrobial study of different ZnO NPs was performed by agar well diffusion method. The relative activities of these samples were studied against both Gram-positive *Staphylococcus aureus (NCIM 2654)* and Gram-negative *Proteus vulgaris (NCIM 2613)*bacteria. In this method, in each well 1 mg/ml concentration of all ZnO NPs was inoculated on nutrient agar plates which were previously seeded by 100 µl of 24 h old bacterial inocula. ZnO samples were sonicated for 15 min in distilled water before inoculation. Then the plates were incubated at 37 °C for 24 h for the growth of microorganisms. Antimicrobial activity was observed by measuring the inhibition zone diameter (mm).

### 2.6 Determination of minimum inhibitory concentration

The determination of minimum inhibitory concentration was performed in sterile Muller –Hinton broth at concentration of nanoparticles ranging from 10 mg to 50 mg/ml against two pathogens Gram positive *Staphylococcus aureus (NCIM 2654)* and Gram negative *Proteus vulgaris(NCIM 2613)*bacteria. The assay was carried out in 96 well plates by using tryptic soy broth medium. In brief, 200 µl volume of tryptic soy medium was added in each well and inoculated with 24 h old 10 µl of bacterial inocula. One well was maintained without addition of nanoparticles, used as a control. The microplates were incubated at 37 °C for 24 h. After incubation OD was recorded at 600 nm. From graph, minimum inhibitory concentration and % of inhibition at each concentration was determined.

### 2.7 Antibiofilm activity

Antibiofilm activity was done by using microtiter plate method. For this, *Staphylococcus aureus (NCIM 2654)* and *Proteus vulgaris (NCIM 2613)* were inoculated in sterile tryptic soy broth and incubated for 24 h at 37 °C. Then samples were centrifuged at 5000 rpm and pellet was suspended in phosphate buffer(pH 7.0) 1 mg/ml stock of all ZnO samples were prepared. In brief, 200 µl medium with known concentrations of ZnO were inoculated with 10 µl of bacterial suspension and incubated for 24 h at 37 °C. After incubation, the wells were drained, washed with phosphate buffer saline(PBS),fixed with cold methanol, and then stained with 1% crystal violet for 30 min. Biofilm formed in wells was resuspended in 30% acetic acid. The intensity of suspension was measured at 570 nm and % of biofilm inhibition was calculated by using equation given below [8].

%ageofbiofilminhibition=OD490incontrol-OD490intreatment/OD490incontrol×100

## 3 Results and discussion

### 3.1 X-ray diffraction studies

**Fig. 1:**
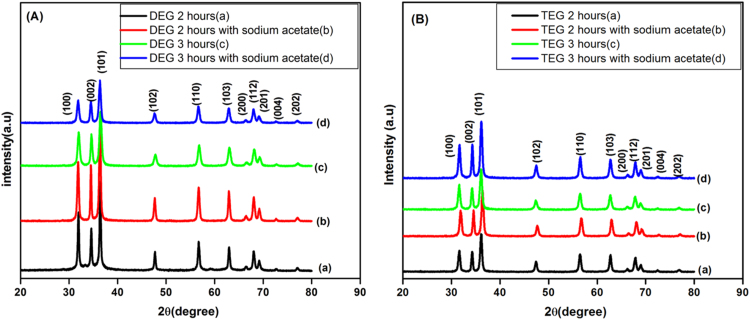
(A) XRD of DEG 2 h(a), DEG 2 h with sodium acetate(b), DEG 3 h(c), DEG 3 h with sodium acetate(d), (B) TEG 2 h(a), TEG 2 h with sodium acetate(b), TEG 3 h(c), TEG 3 h with sodium acetate(d).

Fig. 1 A and B represents diffractograms of ZnO NPS.The XRD of all the samples having 2θ values with reflection planes at 31.72° (100), 34.39° (002), 36.23° (101) and 47.44° (102) corresponds to JCPDS Card No. 36-1451. So,all diffraction peaks fit well with hexagonal wurtzite structure of ZnO, which proves that ZnO was successfully synthesized by polyol hydrolysis method [43].

The crystallite sizes of ZnO NPs were calculated from FWHM of the most intense peak using the Debye–Scherrer formula (Eq. (1)), given below:

(1′) D=0.9λ/βcosφ

### 3.2 UV–vis spectroscopy analysis

**Fig. 2:**
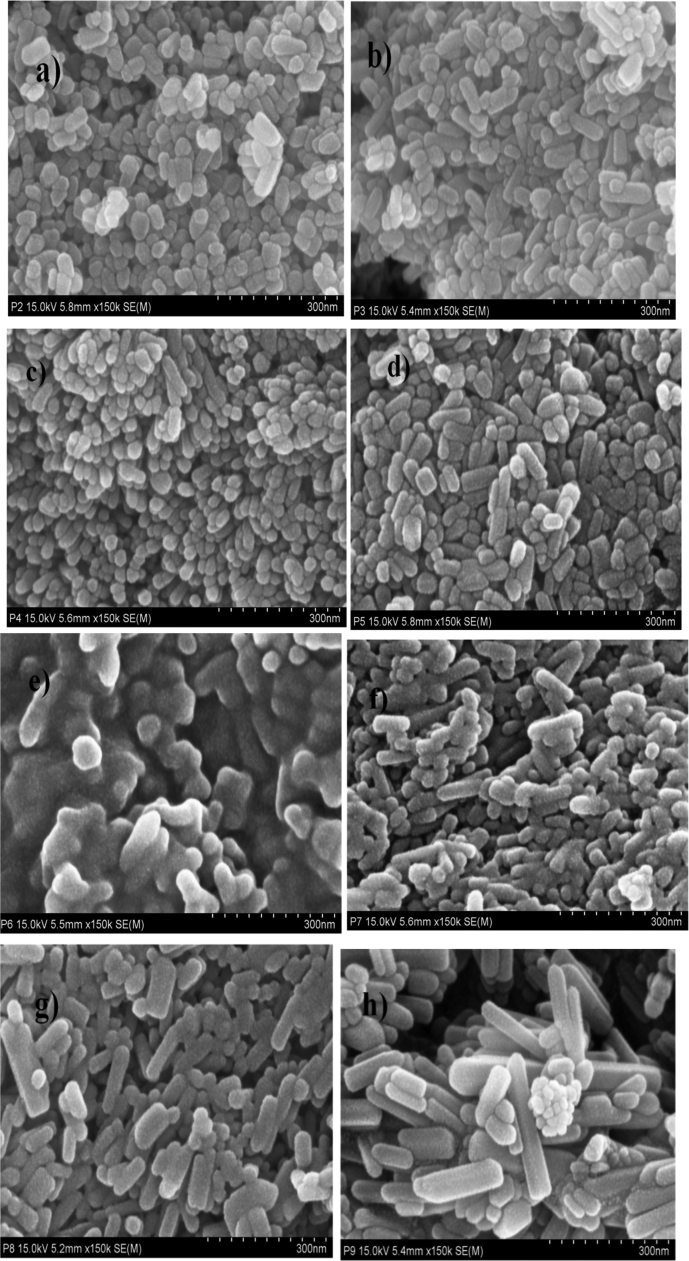
FESEM micrographs of (a) DEG 2 h, (b) DEG 2 with sodium acetate, (c) DEG 3 h, (d) DEG 3 h with sodium acetate, (e)TEG 2 h, (f) TEG 2 h with sodium acetate, (g) TEG 3 h, (h) TEG 3 h with sodium acetate.

**Fig. 3:**
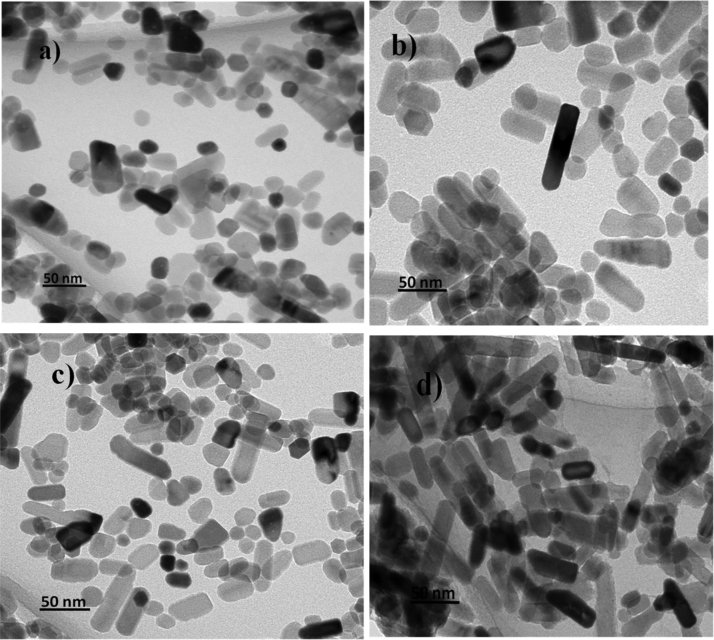
Representative TEM images of (a) DEG 2 h, (b) DEG 2 h with sodium acetate, (c) DEG 3 h, (d) DEG 3 h with sodium acetate, (e)TEG 2 h, (f) TEG 2 h with sodium acetate, (g) TEG 3 h, (h) TEG 3 h with sodium acetate.

In order to observe the UV spectroscopy of synthesized ZnO nanoparticles, they were sonicated in distilled water for about 15 min and UV spectra were recorded Supplementary data Fig. 1 A and B shows the UV–vis absorption spectra of the ZnO nanoparticles synthesized by using DEG and TEG. The absorption peak was recorded in each spectrum in range of 360–380 nm which is a characteristic band for the pure ZnO.Absence of any other peak in the spectrum confirms that the synthesized products are ZnO only [17]. (Fig. 2, Fig. 3).

It is reported that the intensity of absorption peak in UV–visible spectrum is related with particle size of nanoparticles. As the particle size decreases, absorption peak shifts towards lower wavelength that is blue shift. As in case of DEG mediated synthesized ZnO nanoparticles, DEG 2 h sample shows absorption peak at 366 nm while DEG 2 h sample with sodium acetate show absorption peak at 368 nm. Similarly remaining all samples show blue shift with decrease in particle size which interpret that the intensity of the absorbance peak shows slight blue shift with decrease in particle size. The type of polyols used, temperature and reaction time have effect on absorption peak [44], [45].

### 3.3 Field emission scanning microscopy (FESEM)/energy dispersive X-ray spectroscopy (EDX)

**Fig. 4:**
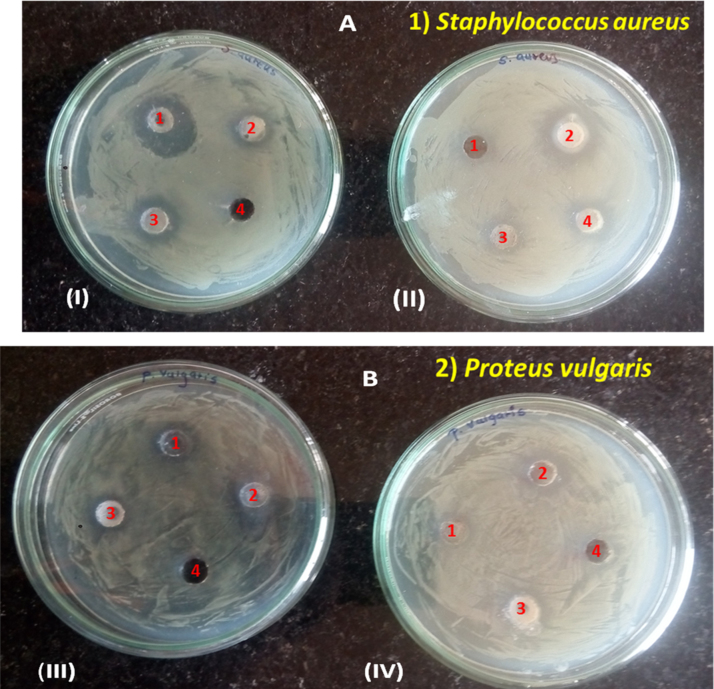
Antibacterial activity of DEG and TEG mediated synthesized ZnO NPs (1 mg/ml) against Gram-positive *Staphylococcus aureus(NCIM 2654)*(A)and Gram-negative *Proteus vulgaris(NCIM 2613)* (B), In plate (I) and (III) samples inoculated are(1)DEG 3 h, (2) DEG 3 h with sodium acetate, (3) DEG 2 h, (4) DEG 2 h with sodium acetate and in plate (II) and (IV) samples inoculated are(1)TEG 2 h with sodium acetate, (2) TEG 3 h, (3) TEG 3 h with sodium acetate, (4) TEG 2 h.

**Fig. 5:**
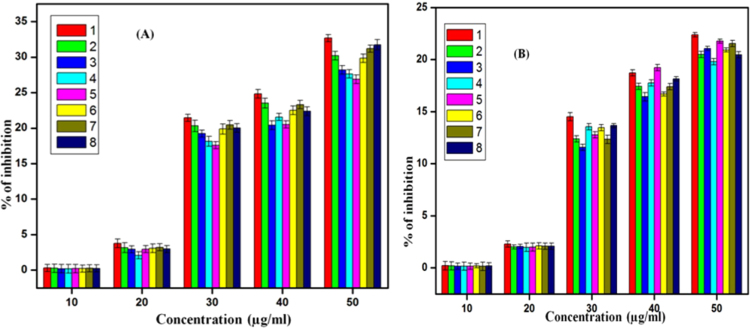
% of inhibition of all ZnO samples at different concentrations of all ZnO nanoparticles against *Staphylococcus aureus(NCIM 2654)*(A) and *Proteus vulgaris(NCIM 2613)*(B), (1) DEG 3 h, (2) DEG 3 h with sodium acetate, (3) TEG 3 h, (4) TEG 3 h with sodium acetate, (5) TEG 2 h, (6)TEG 2 h with sodium acetate, (7) DEG 2 h, (8) DEG 2 h with sodium acetate.

Morphology of all ZnO nanoparticles synthesized by using DEG and TEG were studied by images obtained by FESEM and TEM. Fig. 4, Fig. 5 clearly shows that the zinc oxide nanoparticles obtained by refluxing diethylene glycol and triethylene glycol for 2 h and 3 h in presence and in absence of sodium acetate have uniform shape and size with different morphology. Image depicts addition of sodium acetate, use of different polyol and change in reflux time from 2 h to 3 h offers difference in morphology from oval to rod shape with average particle size of ~ 15 to 100 nm. FESEM and TEM analysis reports DEG refluxed for 3 h in absence of sodium acetate exhibited least particle size of ~ 15 nm.

The difference observed in the morphology of the ZnO nanoparticles depends upon release rate of OH ^–^ ions. In presence of sodium acetate release rate of OH^-^ ions becomes slow due to its weak hydrolyzing ability of acetate ions, which affects on condensation and nucleation process. So particles show elongated rod shaped morphology [38].

The elemental analysis of all ZnO nanostructures was performed by EDX spectroscopy. The Supplementary Fig. 2 shows the EDX of all synthesized ZnO nanoparticles which reveals presence Zn and O that indicate the synthesis of pure ZnO nanoparticles. The impurity free nanoparticle exhibits the promising anti-microbial and antibiofilm activity.

### 3.4 Fourier Transform Infrared Spectroscopy (FT-IR) analysis

In Supplementary data Fig. 3 A and B, FTIR spectrum of ZnO nanoparticles synthesized in DEG and TEG showed characteristic peak at ~ 3443 cm^−1^, which was assigned to stretching vibrations of hydroxyl group [46], [47] and the peaks at ~ 2922 cm^−1^ were assigned to –CH stretching showing presence of CH_2_,CH_3_ groups [48]. The 2 peaks at about ~ 1586 cm ^−1^ and ~ 1412 cm ^−1^ were assigned to symmetric and asymmetric C˭O stretching [49]. The peak position at 1125 cm^−1^ were assigned to –CH deformation showing –CH_2_, CH_3_ bending. Due to inter atomic vibrations, metal oxides generally exhibit absorption bands in fingerprint region below 1000 cm^−1^. [50]. In the infrared region, the peaks at around 415–480 cm^−1^corresponds to ZnO which show the stretching vibration of Zn-O [51]. This observation indicate that, DEG/TEG molecules get adsorbed on synthesized ZnO nanoparticles [48]. The differences in the particle sizes may lead to different wavenumber and frequencies are consistent to the reported literature [52].

### 3.5 Thermogravimetric analysis

The thermal decomposition behaviour and presence of adsorbed polyols of all ZnO samples were observed by TGA analysis. All samples were heated from 50 to 900 °C at the rate of 10 °C/min. The Supplementary data Fig. 4A and B shows the thermal decomposition of DEG and TEG mediated synthesized ZnO nanoparticles respectively. The two successive decompositions were observed in all samples. The initial weight loss observed was due to the evaporation of surface adsorbed water and moisture occurred in range of 145–270 °C [53] and further 2ndstage of decomposition was observed in the range of 452–490 °C due to loss of adsorbed DEG/TEG molecules in all samples and which was confirmed by FTIR [54].

### 3.6 Applications of ZnO NPs

#### 3.6.1 Antimicrobial activity

From the results in Table 4, it was observed that among all ZnO nanoparticles the smallest ZnO nanoparticles synthesized in DEG for 3 h showed significant zone of inhibition against *Staphylococcus aureus(NCIM 2654) and Proteus vulgaris(NCIM 2613).*

**Table 4:** Diameter of zone of inhibition by ZnO against *Staphylococcus aureus and Proteus vulgaris*.

Sample	Zone of inhibition in diameter(in mm)	
	*Staphylococcus aureus*	*Proteus vulgaris*
DEG 3 h	14	6
DEG 3 h with sodium acetate	6	4
DEG 2 h	6	2
DEG 2 h with sodium acetate	1	1
TEG 2 h with sodium acetate	1	1
TEG 3 h	7	4
TEG 3 h with sodium acetate	4	3
TEG 2 h	4	1

The intensity of antibacterial activity is size dependent. Intensity of antibacterial activity is inversely proportional to the size of nanoparticles, so nano-sized ZnO show good antibacterial activity than bulk ZnO [55], [56]. The intensity of inhibition by nanoparticles depends upon small size, shape and large surface area to volume ratio, as it affects on the interaction with membrane of microorganisms. Yamamoto et al reported, study of antibacterial activity of different sized ZnO nanoparticles (10–50 nm), which showed better antimicrobial property than bulk ZnO (2 µm) [57], [58]. According to Pratap et al., ZnO synthesized by using green route *Coriandrum sativum* leaf extract exhibit antibacterial activity at concentration more than 100 mg/ml [59]. Sharmila et al., demonstrated antibacterial activity of ZnO nanoparticles (22–93 nm) synthesized through green route *Bauhinia tomentosa* leaf extract, which showed antibacterial activity against Gram positive and Gram negative bacteria [60]. Several reports suggest that the action of ZnO on bacterial species is due to release of reactive oxygen species (ROS) species and zinc ions. Generated ROS species, that is, hydrogen peroxide (H_2_O_2_), OH^-^(hydroxyl radicals), O_2_^−2^ (peroxide) and zinc ions from ZnO nanoparticles bind to the negative surface of the cell membrane, leading to disruption of the cells followed by leakage of inner cellular material that causes cell death [61].

In the present study, our interest was to synthesize particles with different morphologies and to study their size dependent antibacterial activity. Out of all synthesized ZnO nanoparticles, DEG 3 h sample with least particle size (~ 15 nm) exhibited comparatively remarkable antibacterial activity against both bacteria. It’s small size and it’s high surface area to volume ratio may helped for more interaction with bacterial cell, than other ZnO NPs with greater size, this could be the reason why these nanoparticles exhibited significant antibacterial activity than other synthesized nanoparticles.

##### 3.6.1.1 Quantitative antimicrobial assay

**Fig. 6:**
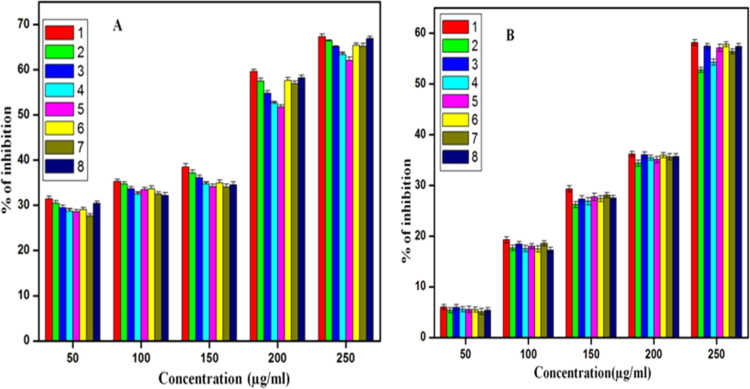
% of biofilm inhibition of all ZnO samples at different concentrations of all ZnO nanoparticles against *Staphylococcus aureus(NCIM 2654)*(A) and *Proteus vulgaris(NCIM 2613)*(B), (1) DEG 3 h, (2) DEG 3 h with sodium acetate, (3) TEG 3 h, (4) TEG 3 h with sodium acetate, (5) TEG 2 h, (6) TEG 2 h with sodium acetate, (7) DEG 2 h, (8) DEG 2 h with sodium acetate.

**Fig. 7:**
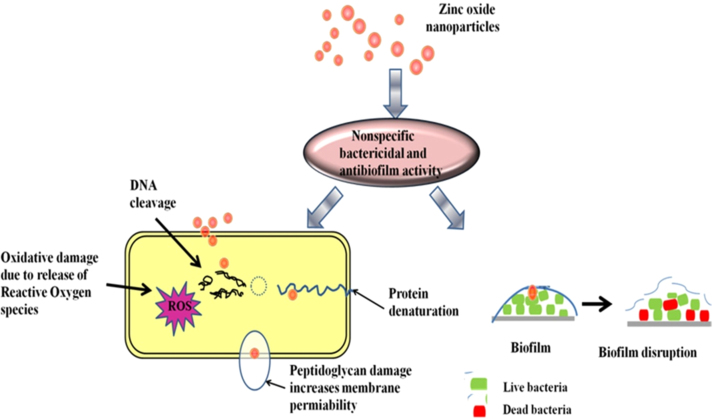
Antibacterial and antibiofilm action of ZnO on bacteria.

From the above results, it was concluded that minimum inhibitory concentration for all samples was in range of 10–20 µg/ml. It was revealed that among all samples DEG 3 h sample showed significant % of inhibition for *Staphylococcus aureus(NCIM 2654)*as compared to *Proteus vulgaris(NCIM 2613).* For *Staphylococcus aureus* and*Proteus vulgaris* it showed 32.67% and 22.38% of inhibition at 50 µg/ml concentration respectively. (Fig. 6, Fig. 7)

##### 3.6.1.2 Antibiofilm activity by microtiter plate

Effect of all synthesized ZnO nanoparticles on biofilm formation on *Staphylococcus aureus (NCIM 2654)* and *Proteus vulgaris(NCIM 2613)* was shown in figure 11 A and B. These graphs indicate that all ZnO samples synthesized by using DEG and TEG inhibited the activity of biofilm formation. Out of all synthesized ZnO nanoparticles, ZnO synthesized by refluxing DEG for 3 h without sodium acetate showed significant % of inhibition in *Staphylococcus aureus* as compared to *Proteus vulgaris* at each concentration. All ZnO samples showed increased % of inhibition with increase in concentration. At 250 µg/ml concentration of ZnO synthesized by DEG refluxed for 3 h exhibited maximum 67.3% and 58.18% biofilm inhibition against *Staphylococcus aureus* and *Proteus vulgaris.*

*Staphylococcus aureus* and *Proteus vulgaris* are pathogens that have ability to form biofilm on medical implants associated with chronic infections. These infections are difficult to irradicate due to resistant nature of biofilm [62]. Action of antimicrobial agents against biofilm associated infections is not that much effective due to inability of penetration into network of biofilm. To overcome this problem application of nanoparticles for inhibition of antibiofilm is efficient [4], [63].

In present study, by using different strategies we have synthesized ZnO nanoparticles with different morphologies in which ZnO nanoparticles synthesized by refluxing DEG for 3 h in absence of sodium acetate proved to be efficient nanoparticle with remarkable antibiofilm activity than other synthesized ZnO nanoparticles with size greater than these particles. These results revealed that smaller nanoparticles exhibited significant inhibition of biofilm than larger nanoparticles.

## 4 Conclusion

In the present investigation, we have synthesized ZnO nanoparticles by applying different approaches, i) regular synthesis in polyols, ii) In presence of sodium acetate, iii) increasing reaction time. We showed that it is possible to control shape and size of nanoparticles through these approaches. XRD analysis revealed the phase purity. The synthesized nanoparticles have crystallite nature having hexagonal wurtzite structure. UV spectroscopy showed that absorption edges was shifted to a shorter wavelength showing blue shift due to decrease in crystal size. FTIR and TGA analysis presented that DEG and TEG molecule adsorbed on ZnO nanoparticles. The prepared all ZnO nanoparticles posses antibacterial and antibiofilm activity against *Staphylococcus aureus* and *Proteus vulgaris.* The most interesting observation found in present study is that, all synthesized nanoparticles showed nicely organized oval and rod shaped morphology with different size. In case of nanoparticles synthesized by using polyol DEG, it was observed that, addition of sodium acetate and increase in reflux time from 2 h to 3 h changes morphology of nanoparticles from oval to rod shape, while in case of nanoparticles synthesized by using polyol TEG all particles show rod shaped morphology and increase in size with addition of sodium acetate and increase in reflux time from 2 h to 3 h which highlights the role of sodium acetate in change of morphology. Out of all particles, ZnO synthesized by refluxing zinc acetate precursor in DEG for 3 h in absence of sodium acetate with particle size ~ 15 nm showed maximum activity against *Staphylococcus aureus* and *Proteus vulgaris* than other synthesized ZnO nanoparticles. This study showed that the antimicrobial and antibiofilm efficacy of ZnO nanoparticles increases with decreasing particle size. We have demonstrated that applying different approaches affects on the size and shape of nanoparticles, these findings provide better understanding of ZnO nanoparticles that can serve as a potential antibacterial and antibiofilm agent in biomedical application.
