# Supplementary material for: Synthesis and characterization of zinc oxide nanoparticles by using polyol chemistry for their antimicrobial and antibiofilm activity
Source: Biochem Biophys Rep. 2018 Dec 12;17:71–80. doi: 10.1016/j.bbrep.2018.11.007 (PMC6295600; doi:10.1016/j.bbrep.2018.11.007)
Supplement: Supplementary file 1 — Supplementary material [file mmc1.docx]

**Statement of conflict of Interest:**

**The authors declare no conflict of interest and all authors aggress with the submission of manuscripts.**

**On behalf of all authors**

**Raghvendra Bohara: Corresponding Author**
