# Supplementary material for: Synthesis and characterization of zinc oxide nanoparticles by using polyol chemistry for their antimicrobial and antibiofilm activity
Source: Biochem Biophys Rep. 2018 Dec 12;17:71–80. doi: 10.1016/j.bbrep.2018.11.007 (PMC6295600; doi:10.1016/j.bbrep.2018.11.007)
Supplement: Supplementary file 2 — Supplementary material [file mmc2.docx]

Synthesis and characterization of zinc oxide nanoparticles by using polyol chemistry for their antimicrobial and antibiofilm activity

Pranjali P.Mahamuni^a^., Pooja M.Patil^a^., Maruti J.Dhanavade^b^., Manohar V.Badiger^c^.,Prem G.Shadija^a^., Abhishek C.Lokhande^d^., Raghvendra A. Bohara^a,e*^

*^a^Centre for Interdisciplinary Research,D.Y.PatilUniversity,Kolhapur.*

*^b^Departmentof Microbiology,Shivaji University, Kolhapur.*

*^c^CSIR- National Chemical Laboratory,Pune.*

*^d^Department of Materials Science and Engineering, Chonnam National University, Gwangju Republic of Korea*

*^e^CURAM, Center for Research in Medical Devices, National University of Ireland Galway, Ireland*

Corresponding Author

Dr. Raghvendra A Bohara

Assistant Professor & Irish Research Council Fellow

D. Y. Patil University, Kolhapur India & National University of Ireland , Galway, Ireland

**Supplementary data**

**Fig. 1** (A)UV of (a)DEG 2 hours, (b)DEG 2 with sodium acetate, (c)DEG 3 hours, (d)DEG 3 hours with sodium acetate, (B)(a)TEG 2hours, (b)TEG 2 hours with sodium acetate, (c)TEG 3 hours, (d) TEG 3 hours with sodium acetate.

**Fig.2** (a)DEG 2 hours, (b)DEG 2 with sodium acetate, (c)DEG 3 hours, (d)DEG 3 hours with sodium acetate, (e)TEG 2hours, (f)TEG 2 hours with sodium acetate, (g)TEG 3 hours, (h) TEG 3 hours with sodium acetate.

**Fig.3** (A) FTIR spectra of DEG mediated ZnO nanoparticles (a)DEG 2 hours, (b)DEG 2 hours with sodium acetate, (c)DEG 3 hours, (d)DEG 3 hours with sodium acetate, (B)TEG mediated ZnO nanoparticles (a)TEG 2 hours, (b)TEG 2 hours with sodium acetate, (c)TEG 3 hours, (d)TEG 3 hours with sodium acetate.

**Fig.4** (A)TGA of(a)DEG 2 hours ,(b)DEG 2 hours with sodium acetate, (c)DEG 3 hours, (d)DEG 3 with sodium acetate,(B)(a)TEG 2 hours, (b)TEG 2 hours with sodium acetate,(c)TEG 3 hours, (d)TEG 3 hours with sodium acetate.


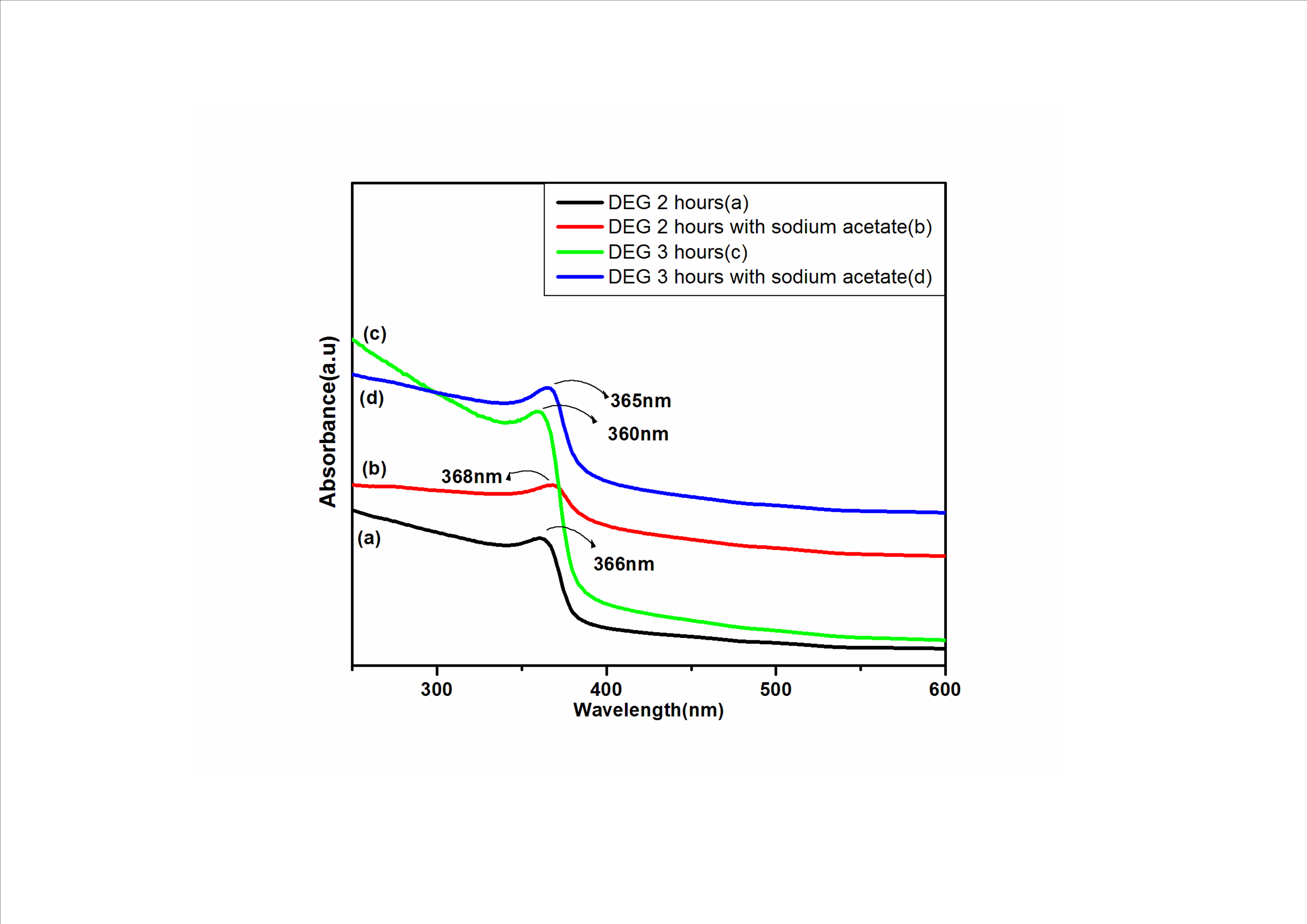


**(A)**


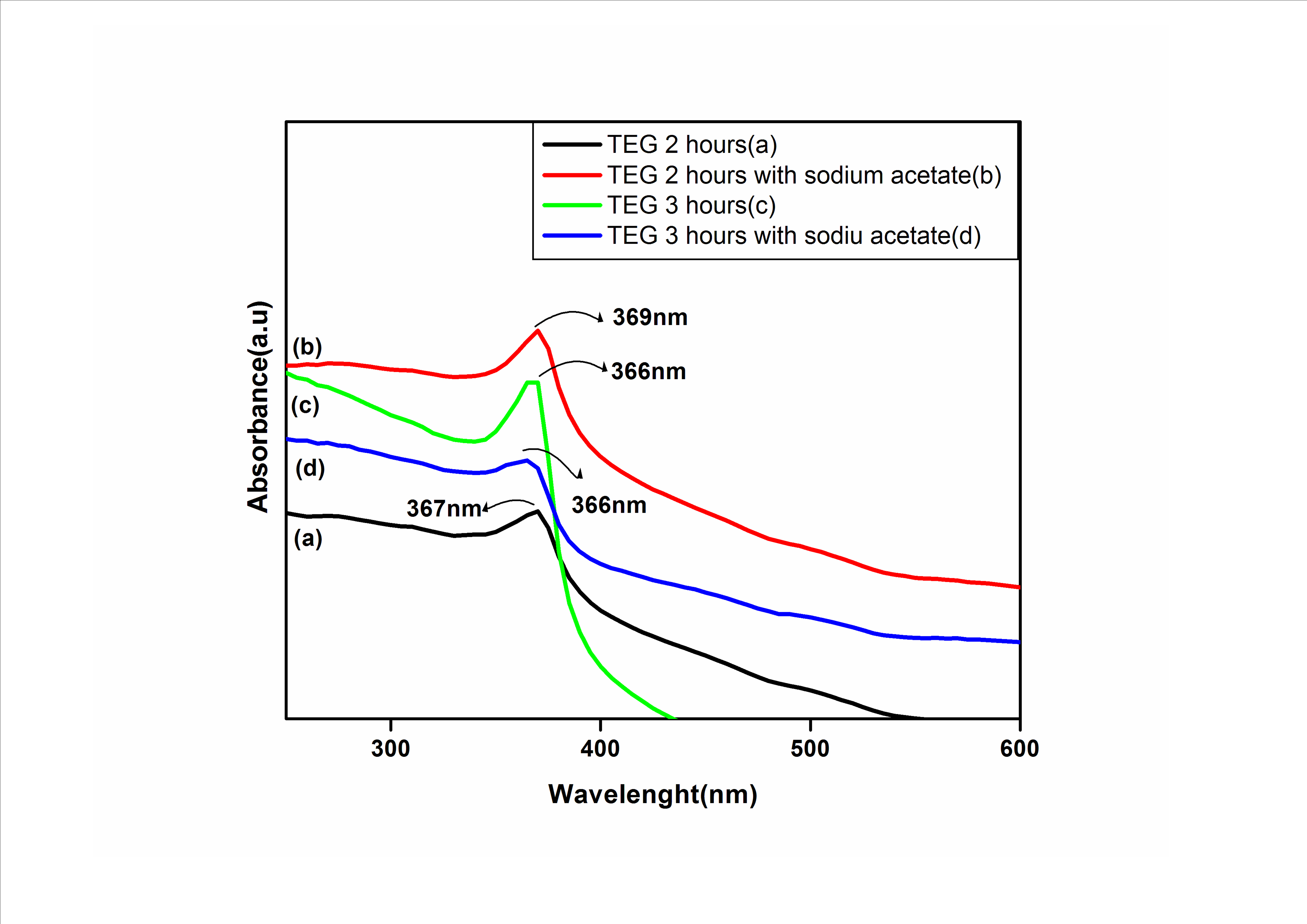


**(B)**

**Fig. 1** (A)UV of (a)DEG 2 hours, (b)DEG 2 with sodium acetate, (c)DEG 3 hours, (d)DEG 3 hours with sodium acetate, (B)(a)TEG 2hours, (b)TEG 2 hours with sodium acetate, (c)TEG 3 hours, (d) TEG 3 hours with sodium acetate.


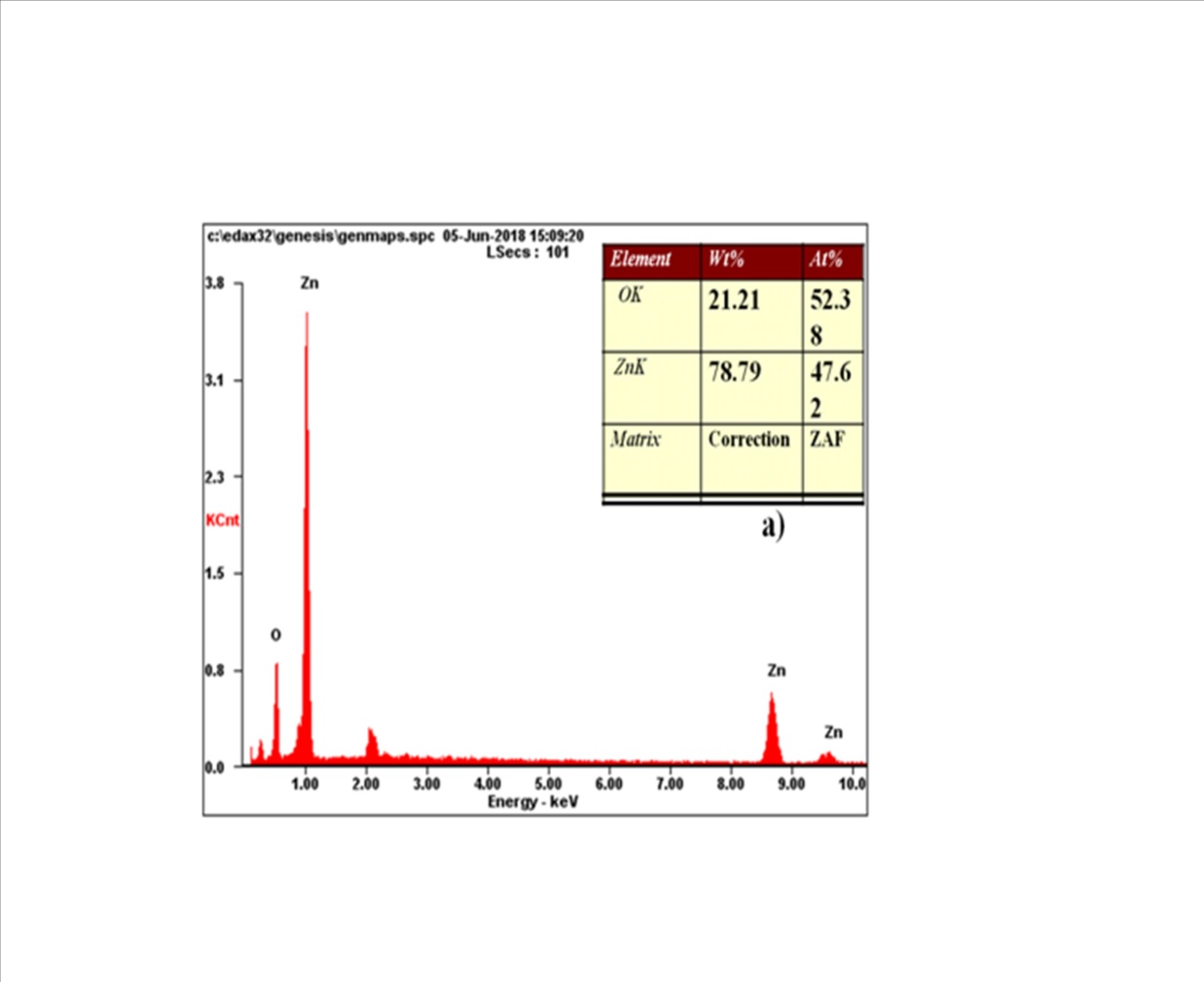

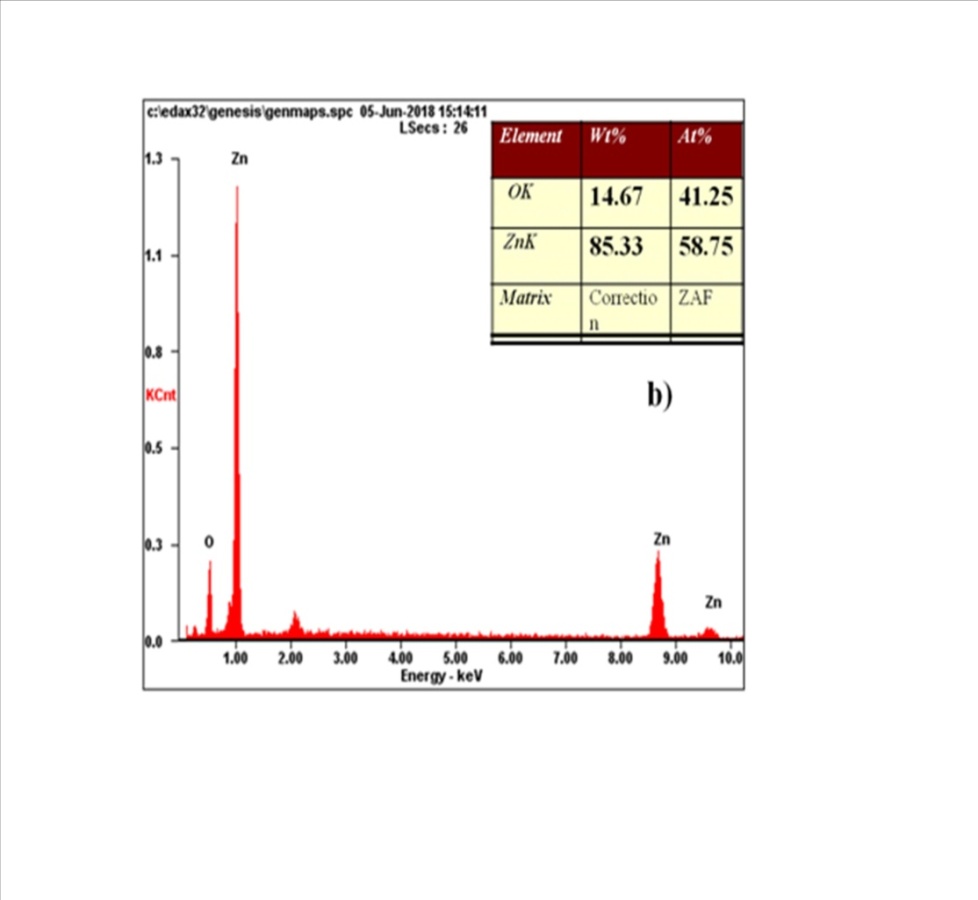


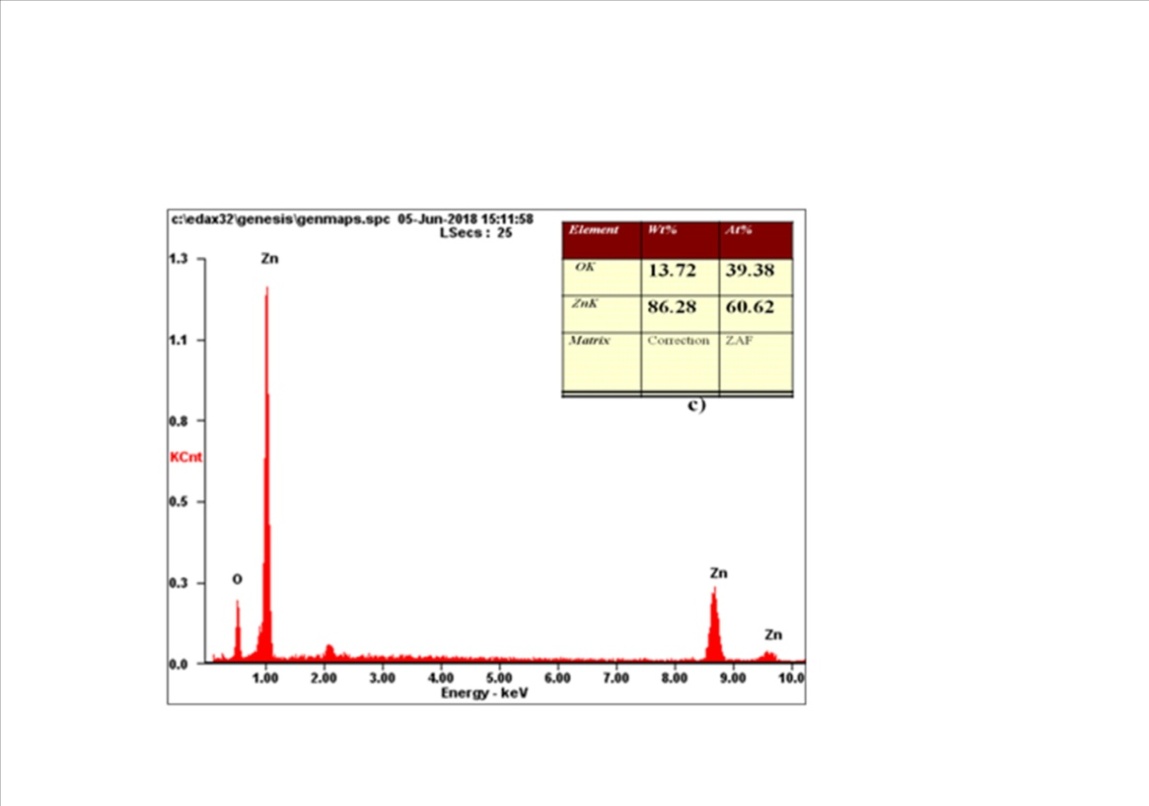

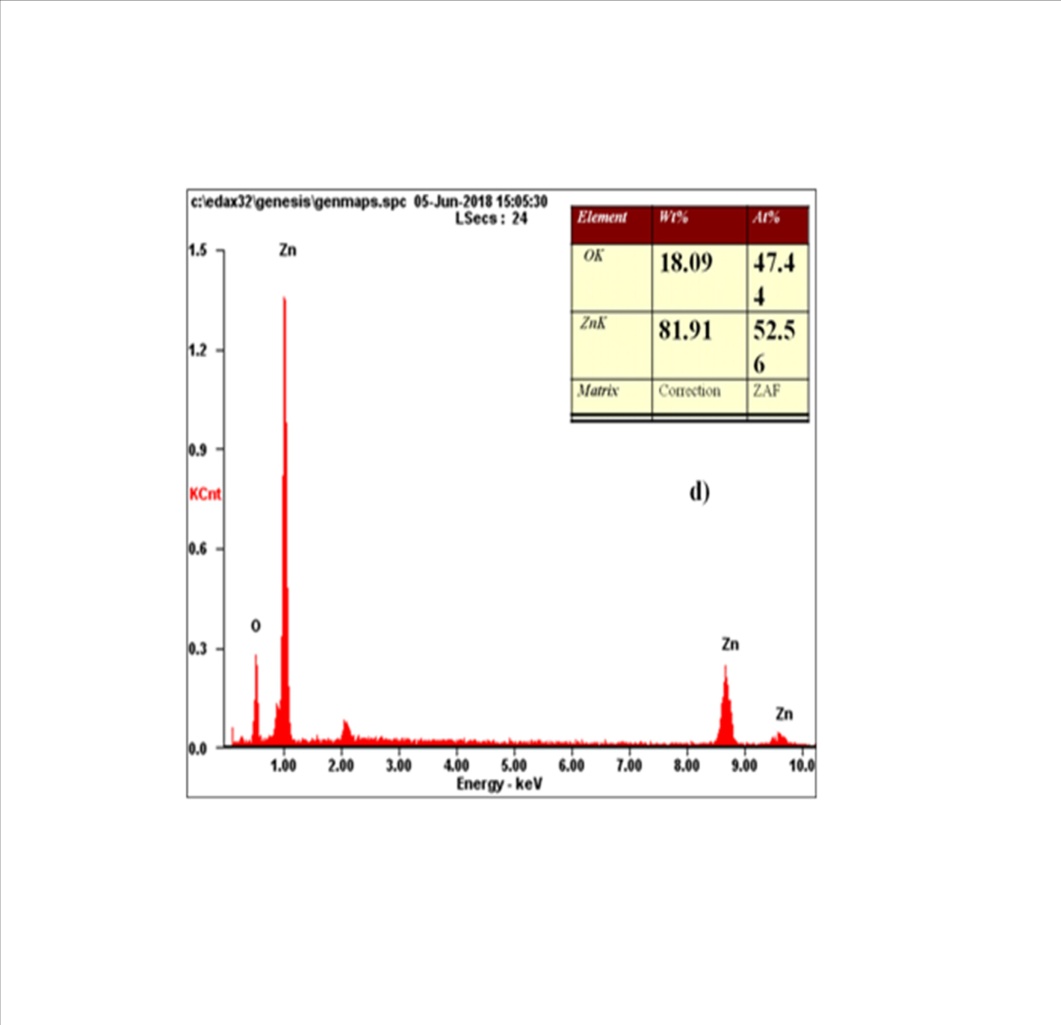


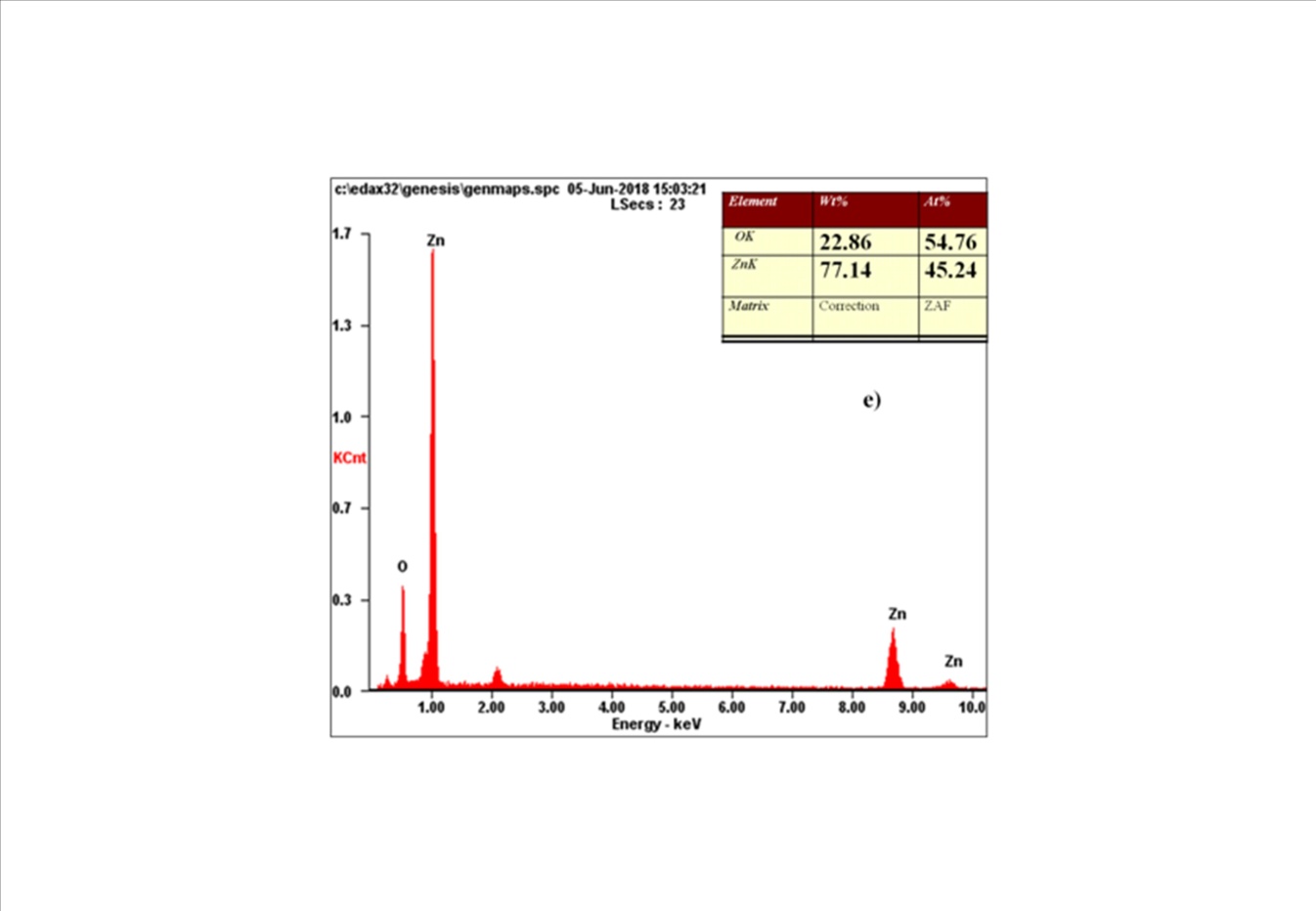

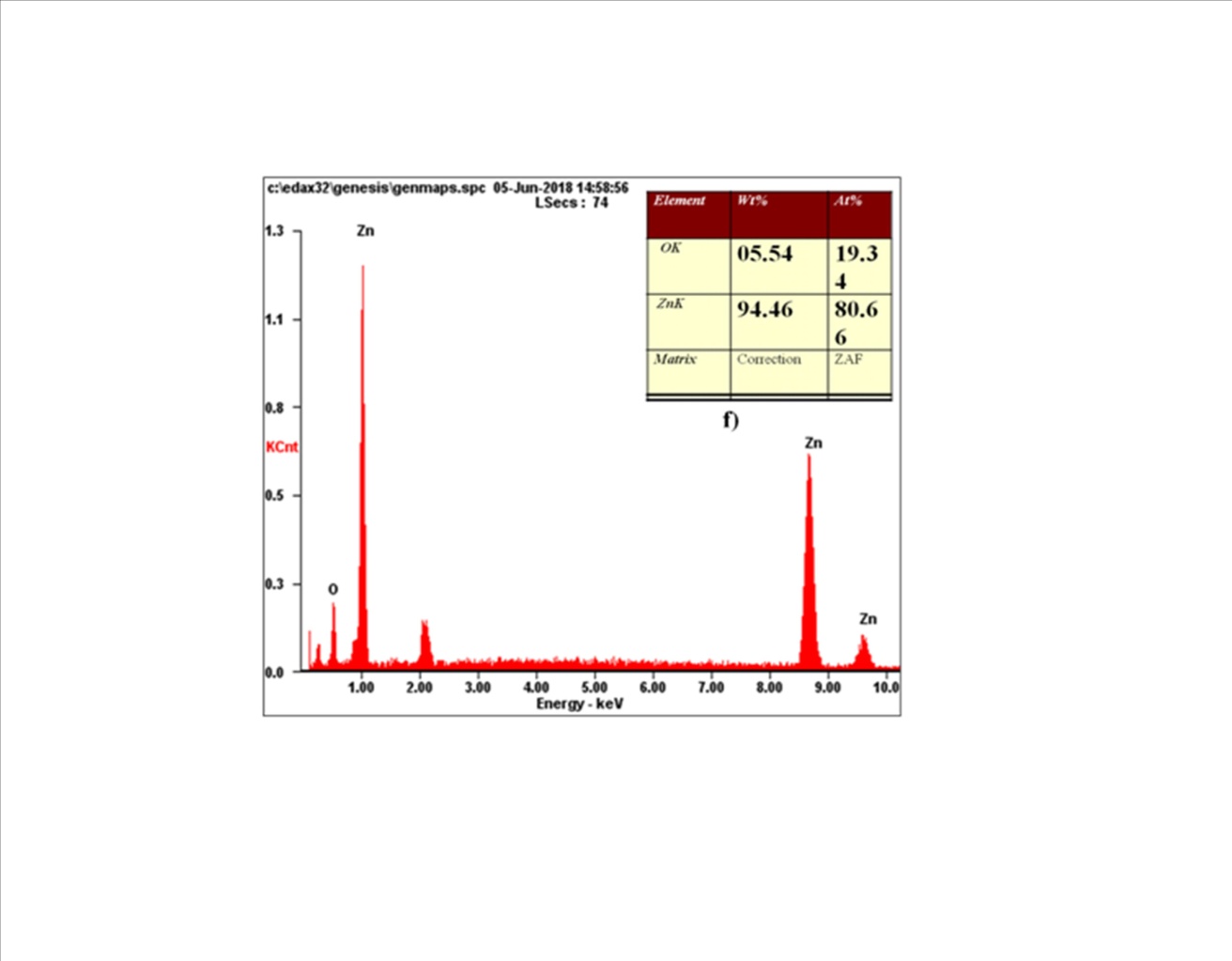


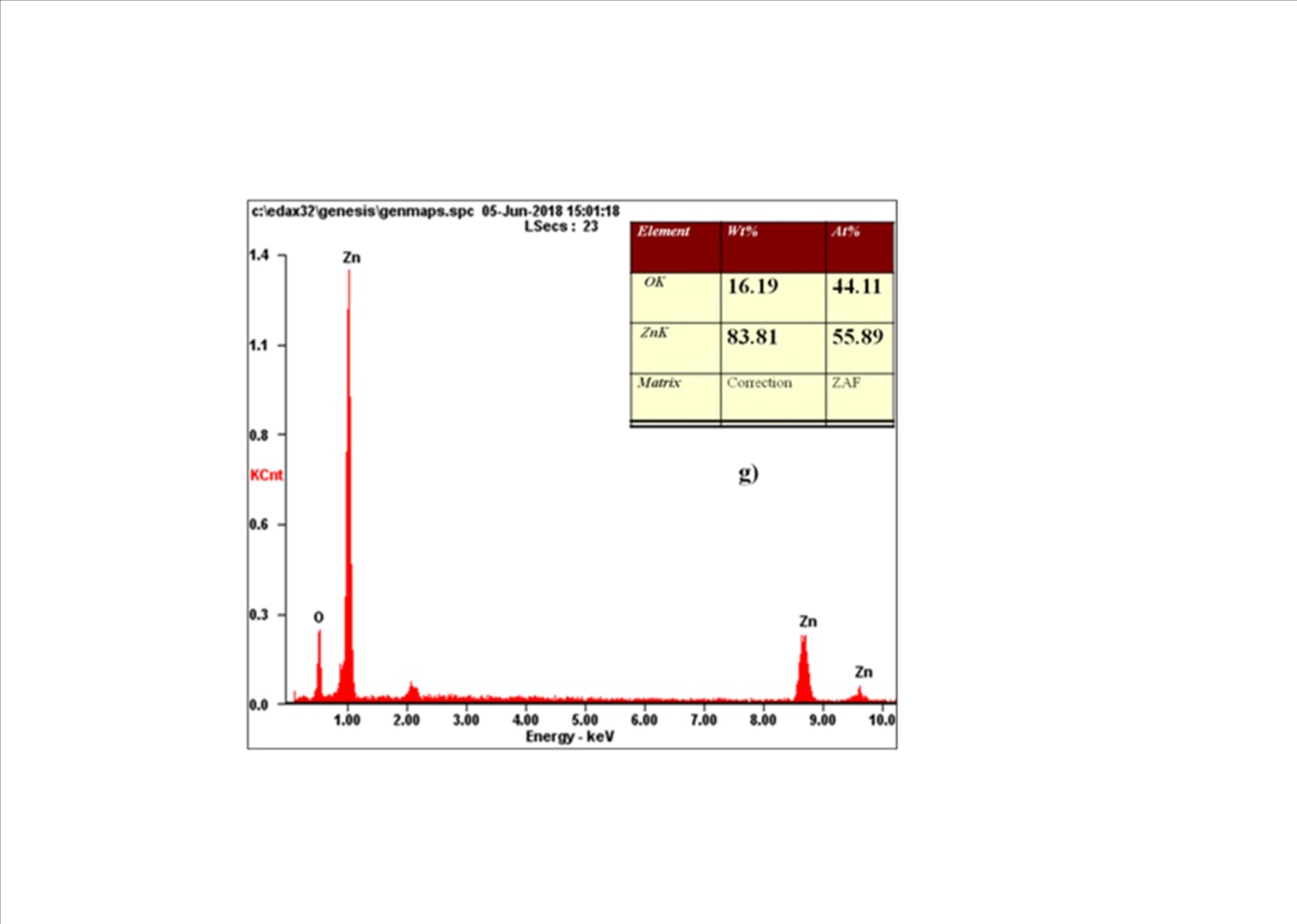

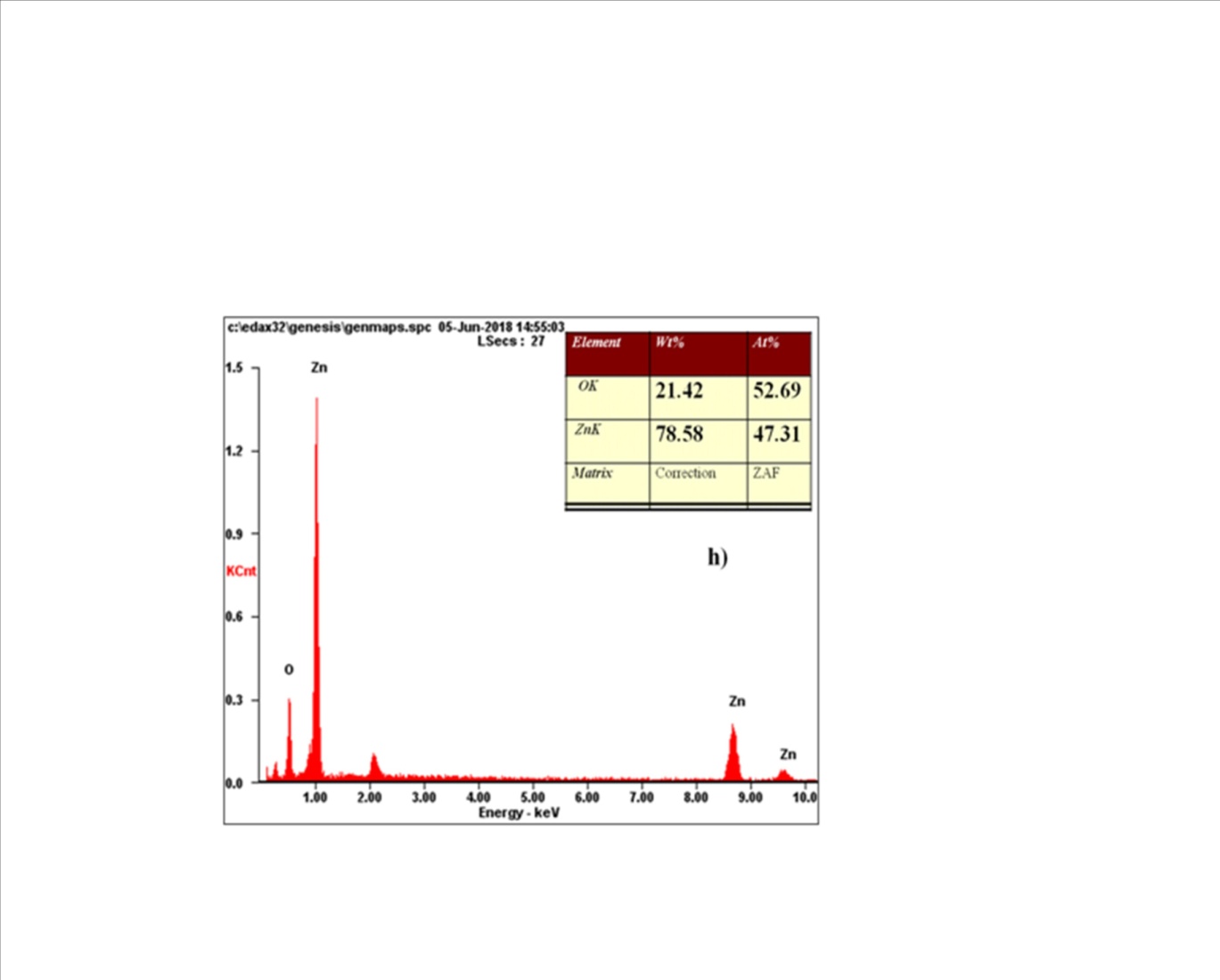


**Fig.2** (a)DEG 2 hours, (b)DEG 2 with sodium acetate, (c)DEG 3 hours, (d)DEG 3 hours with sodium acetate, (e)TEG 2hours, (f)TEG 2 hours with sodium acetate, (g)TEG 3 hours, (h) TEG 3 hours with sodium acetate.


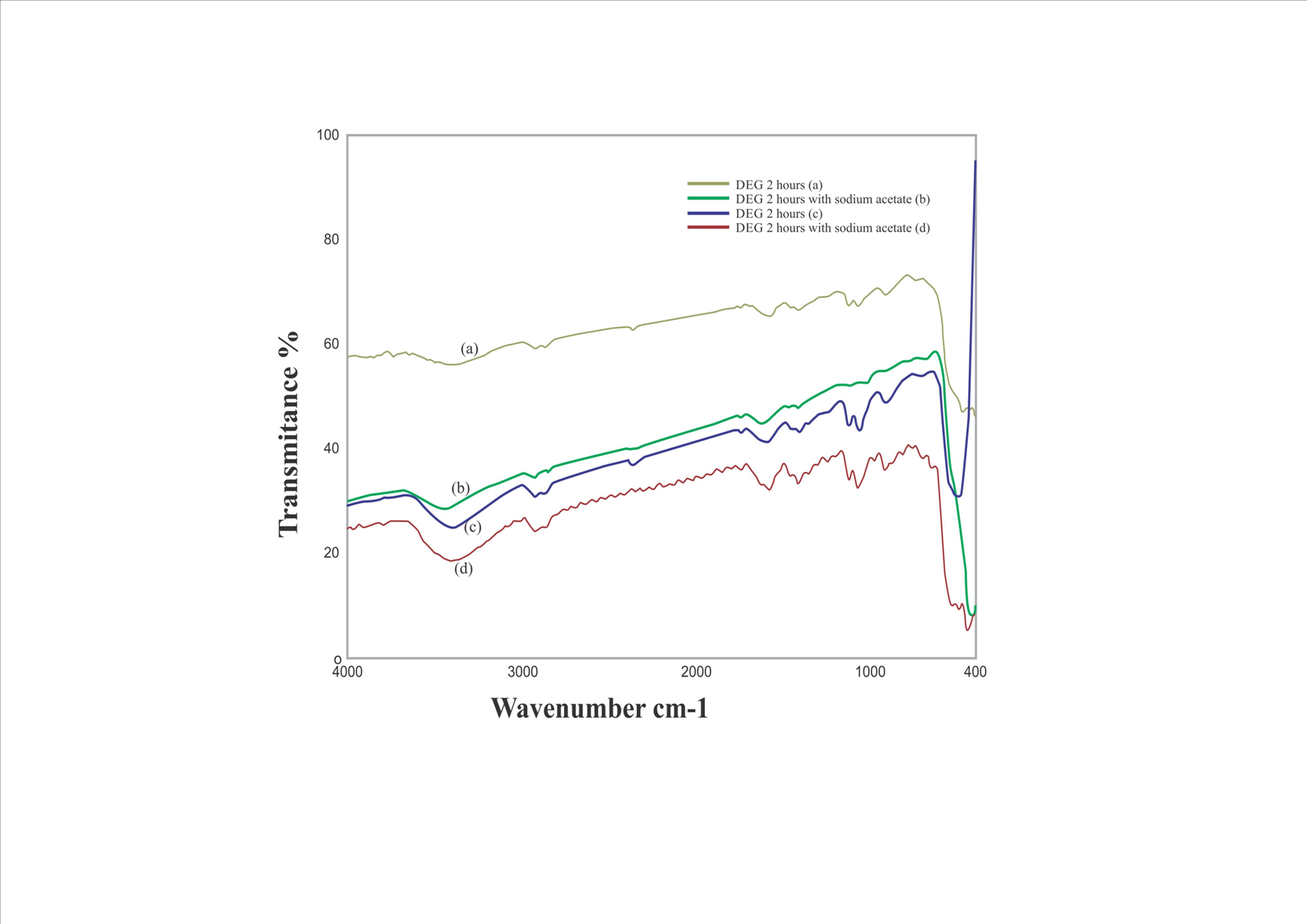


**(A)**


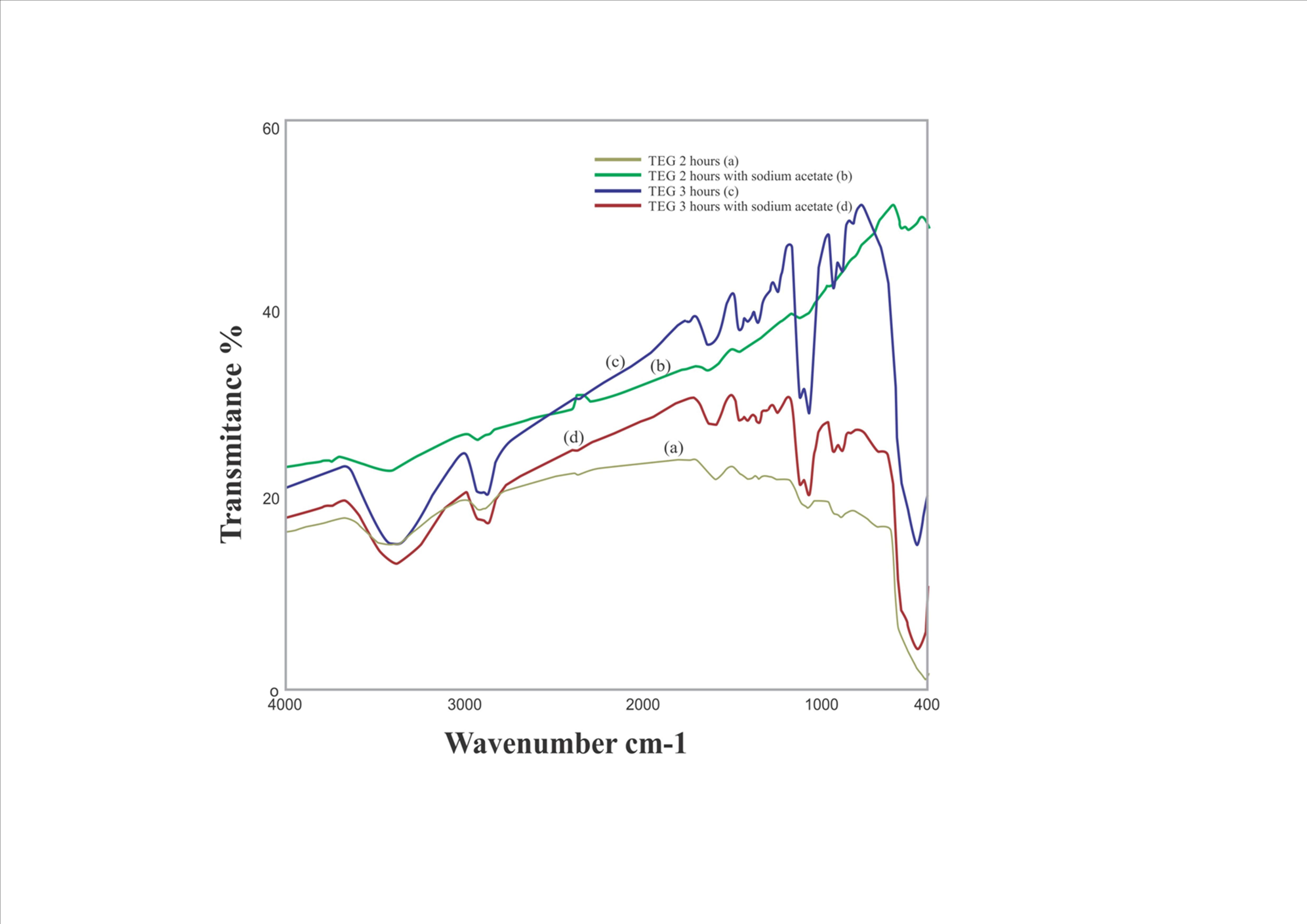


**(B)**

**Fig.3** (A) FTIR spectra of DEG mediated ZnO nanoparticles (a)DEG 2 hours, (b)DEG 2 hours with sodium acetate, (c)DEG 3 hours, (d)DEG 3 hours with sodium acetate, (B)TEG mediated ZnO nanoparticles (a)TEG 2 hours, (b)TEG 2 hours with sodium acetate, (c)TEG 3 hours, (d)TEG 3 hours with sodium acetate.


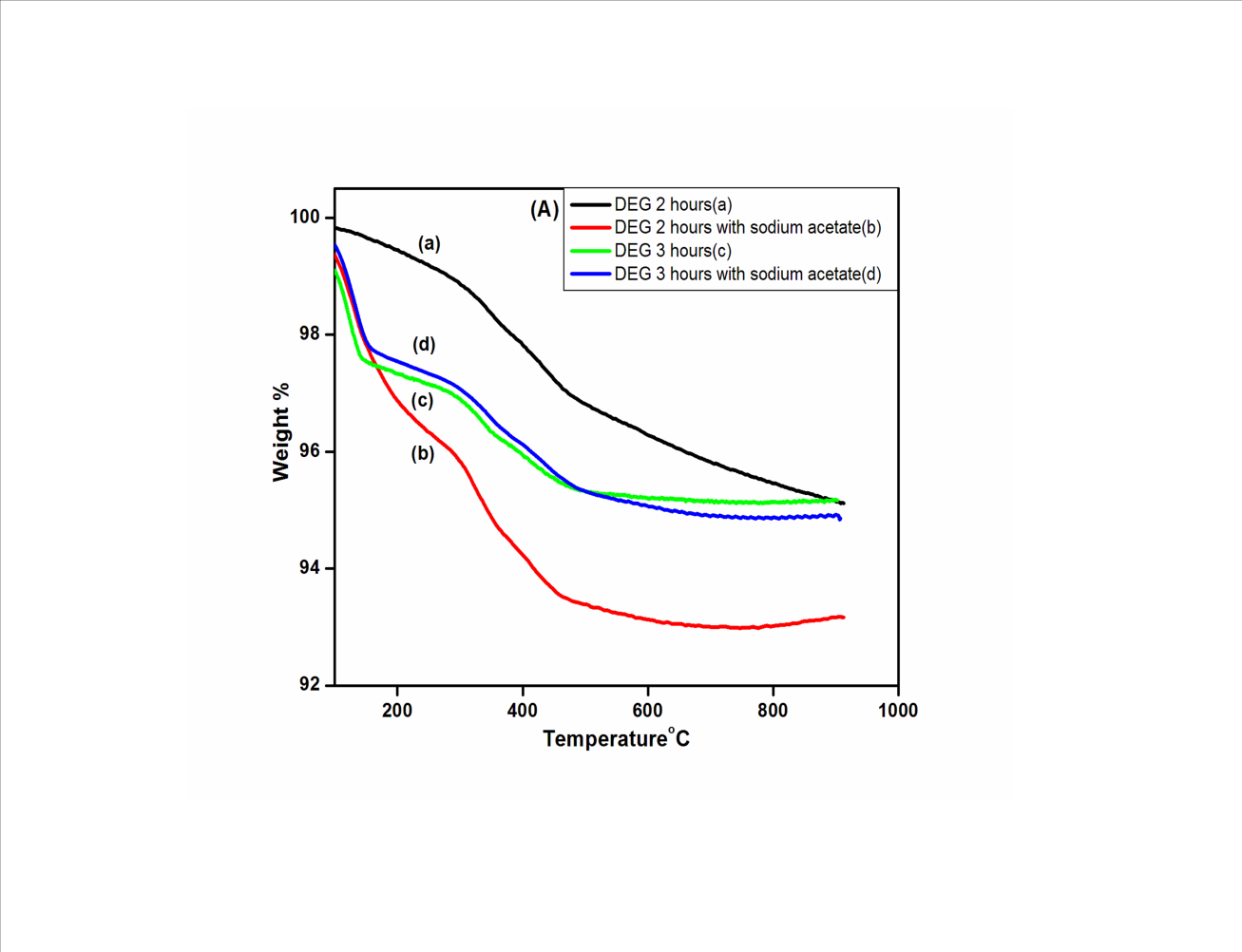

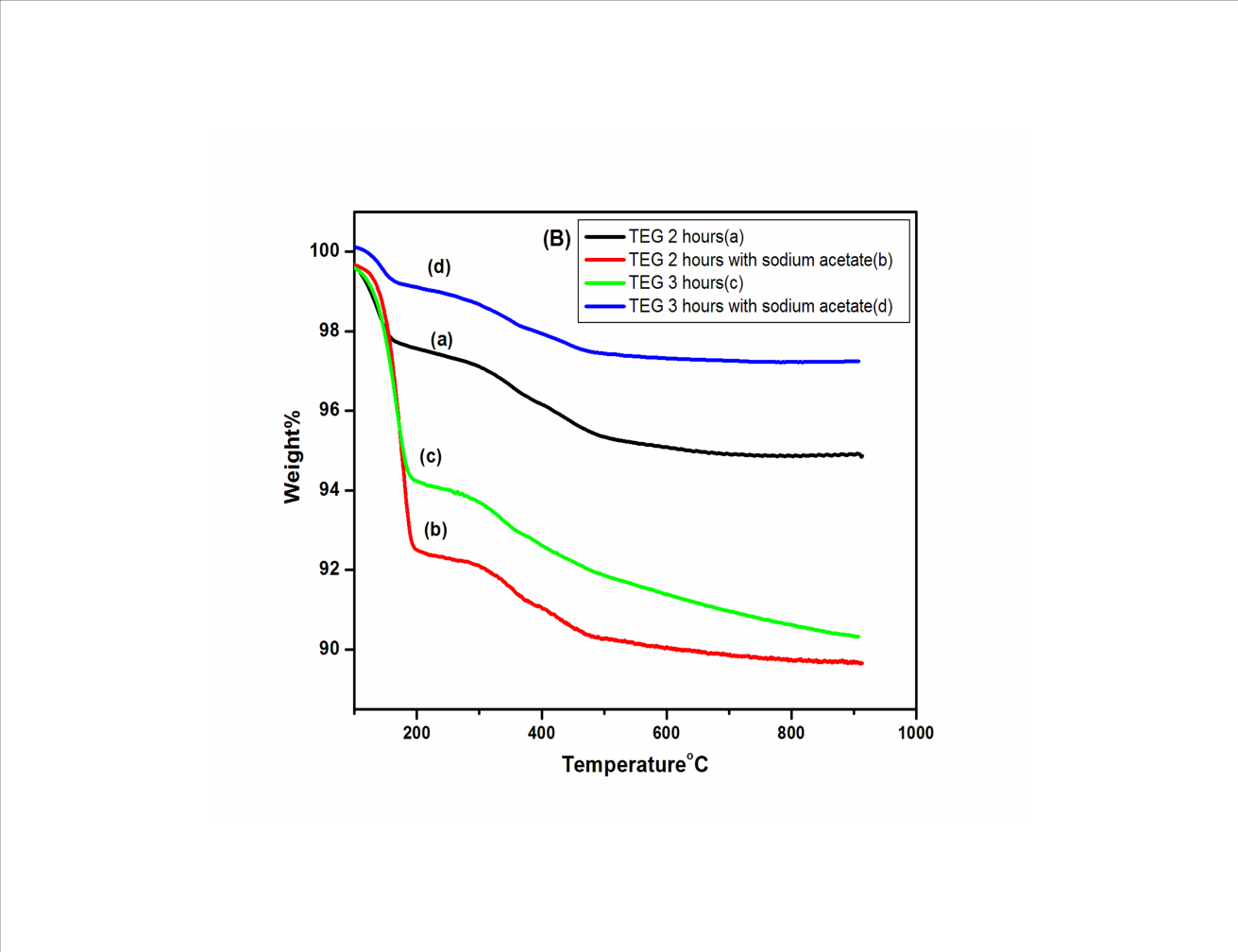


**Fig.4** (A)TGA of(a)DEG 2 hours,(b)DEG 2 hours with sodium acetate, (c)DEG 3 hours, (d)DEG 3 with sodium acetate,(B)(a)TEG 2 hours, (b)TEG 2 hours with sodium acetate,(c)TEG 3 hours, (d)TEG 3 hours with sodium acetate.

**Acknowledgement**

The corresponding author is thankful for D. Y. Patil University for financial support (DYPU/R&D/190) and financial support from the Irish Research Council under the Government of Ireland Postdoctoral fellowship Grant GOIPD/2017/1283. The funding agencies are highly acknowledged.
